# Rethinking the A in FAIR Data: Issues of Data Access and Accessibility in Research

**DOI:** 10.3389/frma.2022.912456

**Published:** 2022-07-27

**Authors:** Hugh Shanahan, Louise Bezuidenhout

**Affiliations:** ^1^Department of Computer Science, Royal Holloway University of London, London, United Kingdom; ^2^DANS (Data Archiving and Networked Services), The Hague, Netherlands

**Keywords:** FAIR, Open Science, data, low/middle income countries, accessibility

## Abstract

The FAIR data principles are rapidly becoming a standard through which to assess responsible and reproducible research. In contrast to the requirements associated with the Interoperability principle, the requirements associated with the Accessibility principle are often assumed to be relatively straightforward to implement. Indeed, a variety of different tools assessing FAIR rely on the data being deposited in a trustworthy digital repository. In this paper we note that there is an implicit assumption that access to a repository is independent of where the user is geographically located. Using a virtual personal network (VPN) service we find that access to a set of web sites that underpin Open Science is variable from a set of 14 countries; either through connectivity issues (i.e., connections to download HTML being dropped) or through direct blocking (i.e., web servers sending 403 error codes). Many of the countries included in this study are already marginalized from Open Science discussions due to political issues or infrastructural challenges. This study clearly indicates that access to FAIR data resources is influenced by a range of geo-political factors. Given the volatile nature of politics and the slow pace of infrastructural investment, this is likely to continue to be an issue and indeed may grow. We propose that it is essential for discussions and implementations of FAIR to include awareness of these issues of accessibility. Without this awareness, the expansion of FAIR data may unintentionally reinforce current access inequities and research inequalities around the globe.

## Fair Data: The New Cornerstone of Responsible Research

Since their conception in January 2016, the FAIR data principles (Wilkinson et al., 2016) have rapidly gained traction and widespread global acceptance. The FAIR data principles were first published under FORCE11^1^ and advocate for the Findability, Accessibility, Interoperability and Reusability of research data and scholarly digital objects more generally. FAIR consists of 15 requirements grouped under the four categories. These requirements serve to guide the actions of data publishers, stewards and other stakeholders to enable responsible data sharing. Central to the concept of FAIR is its application “to both human-driven and machine-driven activities,” with a goal of machine-actionability to the highest degree possible or appropriate. The widespread uptake of the FAIR principles has led to a plethora of diverse activities, including infrastructure development, disciplinary standard setting and ontology creation, and capacity building in data stewardship (Gaiarin et al., 2021). There has been very recent further development in other principles such as the TRUST principles on how repositories should be run (Lin et al., 2020). An analysis of the other principles is beyond the scope of this paper.

The FAIR data standards are an important element of the Open Research ecosystem. Indeed, Open Data, FAIR, and research data management (RDM) are three overlapping but distinct concepts, each emphasizing different aspects of handling and sharing research data (Higman et al., 2019). Higman et al. (2019, p. 1) clarify this relationship in the following way: “FAIR and open both focus on data sharing, ensuring content is made available in ways that promote access and reuse. Data management by contrast is about the stewardship of data from the point of conception onwards. It makes no assumptions about access, but is essential if data are to be meaningful to others.”

Within Open Research, FAIR, Open, and RDM are central not only to practical discussions on infrastructure evolution, but also underpin motivational and aspirational discourse. The ethical drivers of equitable access, transparency as well as the elimination of financial barriers to research outputs play an important role in the evolving aspiration of a “global knowledge commons.” This concept, first introduced by Hess and Ostrom, refers to information, data, and content that is collectively owned and managed by a community of users, particularly over the Internet. Key to the structure of the commons is shared access to digital resources (Hess and Ostrom, 2006, 2007), which emphasizes the reusability—and thus the FAIRness—of data.

While the FAIR data principles have gained rapid acceptance and support, the processes, practices, technical implementation and infrastructures necessary to make data FAIR continue to evolve. It is recognized that realizing a FAIR ecosystem will involve developing key data services that are needed to support FAIR. These include “services that provide persistent identifiers, metadata specifications, stewardship and repositories, actionable policies and Data Management Plans. Registries are needed to catalog the different services” (Collins et al., 2018, p. 8). The challenges of embedding FAIR data practices within research thus include both the technical challenges of creating FAIR-enabling data infrastructures and the need for education and capacity building within research communities.

### Accessibility as a FAIR Principle

The FAIR accessibility principle can be understood as requiring that data are stored properly—for long term—so that it can easily be accessed and/or downloaded with well-defined access conditions. At a minimum, this principle requires access to the metadata. The principle makes a number of requirements of the metadata that accompanies data, including that (A1) (meta)data are retrievable by their identifier using a standardized communications protocol. This includes that (A1.1) the protocol is open, free and universally implementable and that (A1.2) the protocol allows for an authentication and authorization procedure where necessary. It also requires that (A2) metadata are accessible, even when the data are no longer available. In practice this requires that the metadata accompanying the data be understandable to humans and machines, are registered or indexed in a searchable resource and are deposited in a trusted repository (Wilkinson et al., 2016).

As can be seen from the requirements, the FAIR accessibility requirements are highly dependent on the availability of trusted digital repositories and FAIR-oriented curation processes. At the moment the international repository landscape is rapidly evolving, and considerable efforts are being made to promote certification processes to promote FAIR data practices. Indeed, a recent collaboration between the FAIRsFAIR research consortium^2^ and CoreTrustSeal^3^ has worked to integrate FAIR-enabling assessment into the CoreTrustSeal certification of repositories. Integral to this work is the recognition that: “the FAIR Principles are clarified through indicators and evaluated through (ideally automated) tests against digital objects”^4^.

In response to the recognized need for more automated tests for FAIR, a number of data assessment methods and tools have been developed to assign “FAIR scores” to datasets based on a number of criteria. These include automated tools such as F-UJI^5^, FAIR-Enough^6^ and FAIR-Checker^7^, as well as manual and educational tools such as the ARDC FAIR self-assessment tool^8^, and FAIR Aware^9^.

The use of these different assessment tools offers researchers an opportunity to test the FAIR-ness of their data. The scores returned by these tools do vary, according to the criteria included in their design. Nonetheless, regardless of the tool the assessments of accessibility are largely interlinked to the existence of repository and curation infrastructures. For instance, F-UJI scores the accessibility principle on three criteria, namely:

A1-01: metadata contains access level and access conditions of the data
A1-02: metadata is accessible through a standardized communication protocol
A1-03: data is accessible through a standardized communication protocol

When these accessibility requirements are scrutinized, however, it becomes apparent that the FAIR scores returned for any database aim to provide an objective view of access. Within this aim, however, there is an implicit assumption that access is considered solely in relation to the structure of the metadata or data and is independent of the user attempting to access those resources. As a result, the scores cannot be taken to measure the actual accessibility of data or metadata from a *user* perspective. This observation is linked to the realization that depending on the geographic location of the user request the availability of the metadata/data may vary considerably which raises considerable questions. Most pertinent, it becomes important to question whether assigning a FAIR accessibility score to metadata/data could create a false sense of access that undermines existing discussions about inequity in Open Science (Bezuidenhout et al., 2017b; Ross-Hellauer et al., 2022).

## Accessibility and Infrastructures

**Figure 1:**
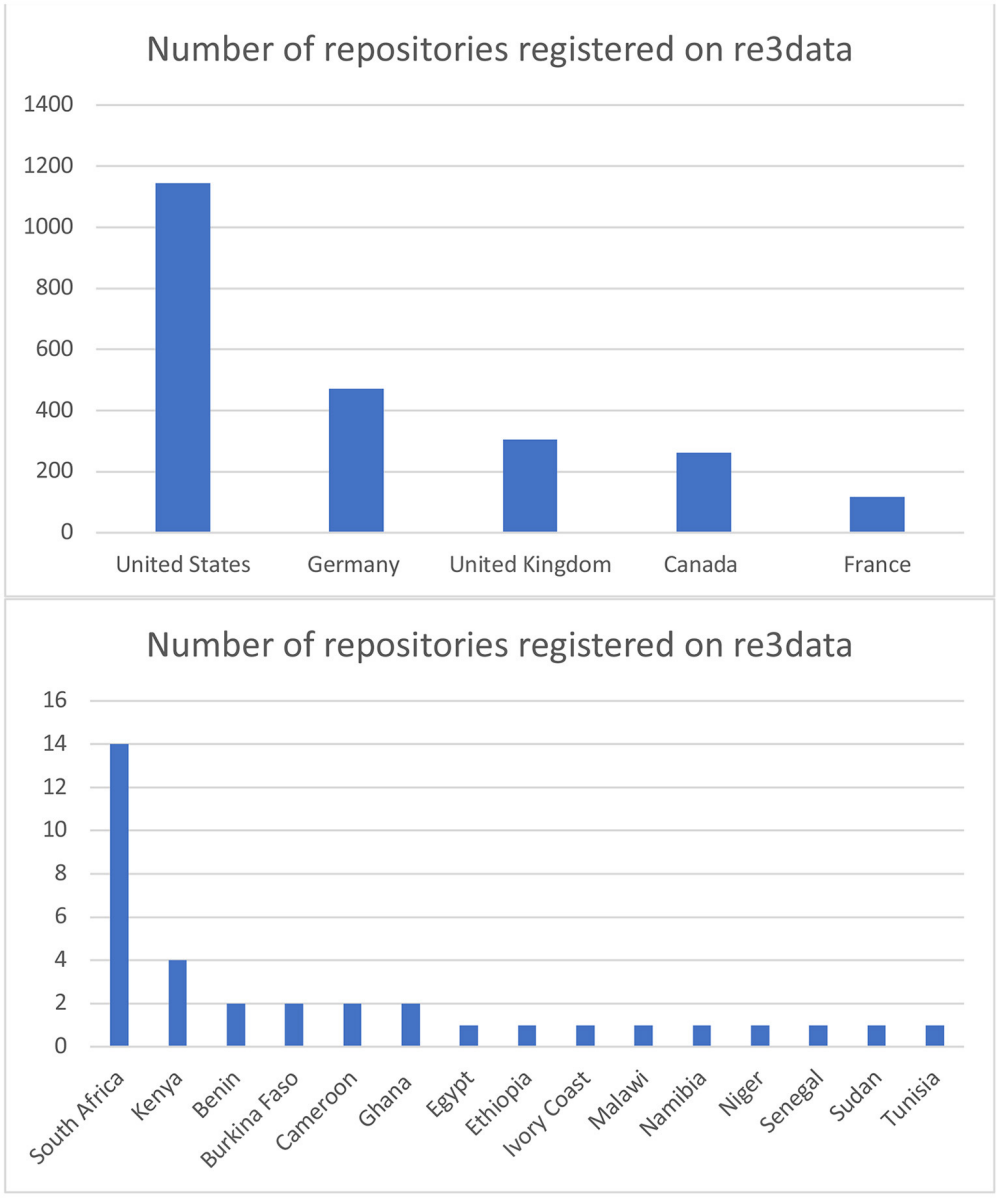
Distribution of repositories within Re3Data according to geographic location in 2022. Available online at: https://www.re3data.org/browse/by-country/ (accessed April 4, 2021).

The European Commission Open Science Monitor tracks trends for open access, collaborative and transparent research across countries and disciplines^10^. The most recent version included a breakdown of the geographic location of the trusted data repositories included in the re3data catalog^11^. As is evident in Figure 1 below, a high number of the data repositories (2,299) registered on re3data reside in just five countries: USA, Germany, UK, Canada, and France. Similarly, many other high-income countries (HICs) host multiple repositories.

In contrast, the whole of the African continent has 35 repositories registered on re3data. Aside from Kenya (4) and South Africa (14), all other countries host either one or two^12^. This unequal global distribution of repositories contributes to the accessibility concerns outlined in the section above. These concerns group around two key issues, namely geopolitical and infrastructural access problems. These concerns are discussed in more detail below.

### Geoblocking and Access Restrictions

Geoblocking is a term used to describe the intentional blocking or restriction of access to websites, apps or other internet content depending on the geographic location of the users. Geoblocking is commonly used in commercial applications to segment customers geographically, and often goes largely unnoticed within the general research community. Indeed, the 2018 ban on geoblocking between Member States of the European Union has even further lowered the visibility of this topic^13^.

**Figure 2:**
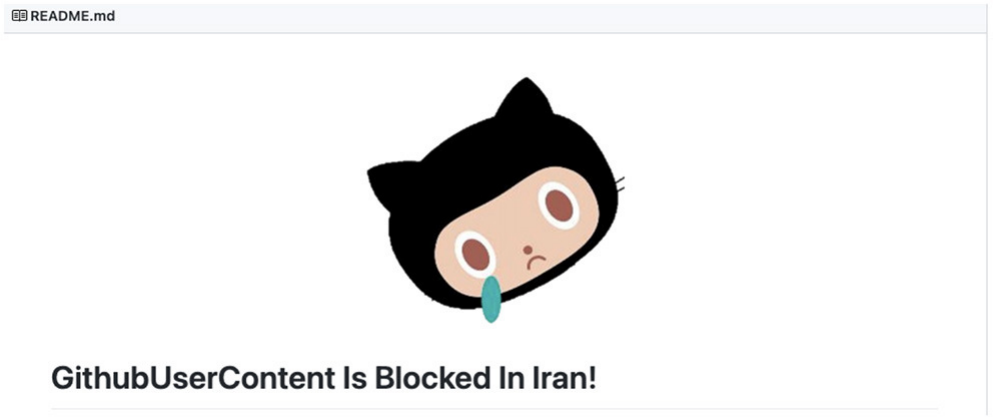
User message returned from a GitHub access request by Iranian users in 2020. Available online at: https://github.com/pi0/github-is-blocked-in-iran (accessed April 4, 2021).

In recent years, however, a small number of academic studies have drawn attention to the impact of geoblocking practices on research (Bezuidenhout et al., 2019; Bezuidenhout and Havemann, 2021). A key observation from these studies is that the Open Science ecosystem is increasingly being populated by diverse actors and many commercial companies are offering key services to the research community. As commercial companies, these actors are subject to the financial legislation of the country in which they are registered. For commercial companies registered in the USA, for instance, this means that they are prohibited from transacting with customers/users residing within countries against which the USA holds financial sanctions. As evidenced in Figure 2 below, the US financial sanctions in place against Iran means that Iranian researchers were unable to access GitHub, a key Open Science tool until 2021^14^. Researchers in Syria and Crimea continue to experience access blocks to GitHub.

Geoblocking on web sites bars access to web sites on the basis of the country a user is sending a request for a web page (identified with a Uniform Resource Location, URL) through their web browser. It is important to note that even when a web site blocks a user both the browser and web site will for the most part exchange some data (unless a web site is not responsive if it is down or because there are connectivity issues). Web standards for geolocation exist^15^ but they are based on the user providing additional data such as their longitude and latitude. A simpler method for identifying a user's country is through the Internet Protocol (IP) address the browser is sending the request from. Web server software, such as Apache HTTP server,^16^ allow web developers to control access to either a particular directory or whole site with a suitably configured file to block access through this IP based approach. In this case, a web browser will receive from the web site a specific error code, namely a 403 code^17^. As repositories will have limited resources, it is unlikely that they will develop more sophisticated approaches to geoblock users.

Similarly, the difficulties of conducting financial transactions from countries under sanctions makes it extremely difficult for researchers within these sanctioned countries to engage with key research activities. These include publishing in academic journals, paying membership fees to academic societies or membership-dependent resources, and buying key software/hardware to refine their datasets (Adam, 2019; Bezuidenhout et al., 2019). These issues of access also extend other key data repositories and collections. Through discussions within dual-use and biosecurity communities it is certain that access to datasets and data tools can be restricted according to the geographic location of the users. This includes, for example, reports of the USA blocking Chinese supercomputer groups^18^, and the USA restricting access to climate change data due to security concerns^19^.

**Figure 3:**
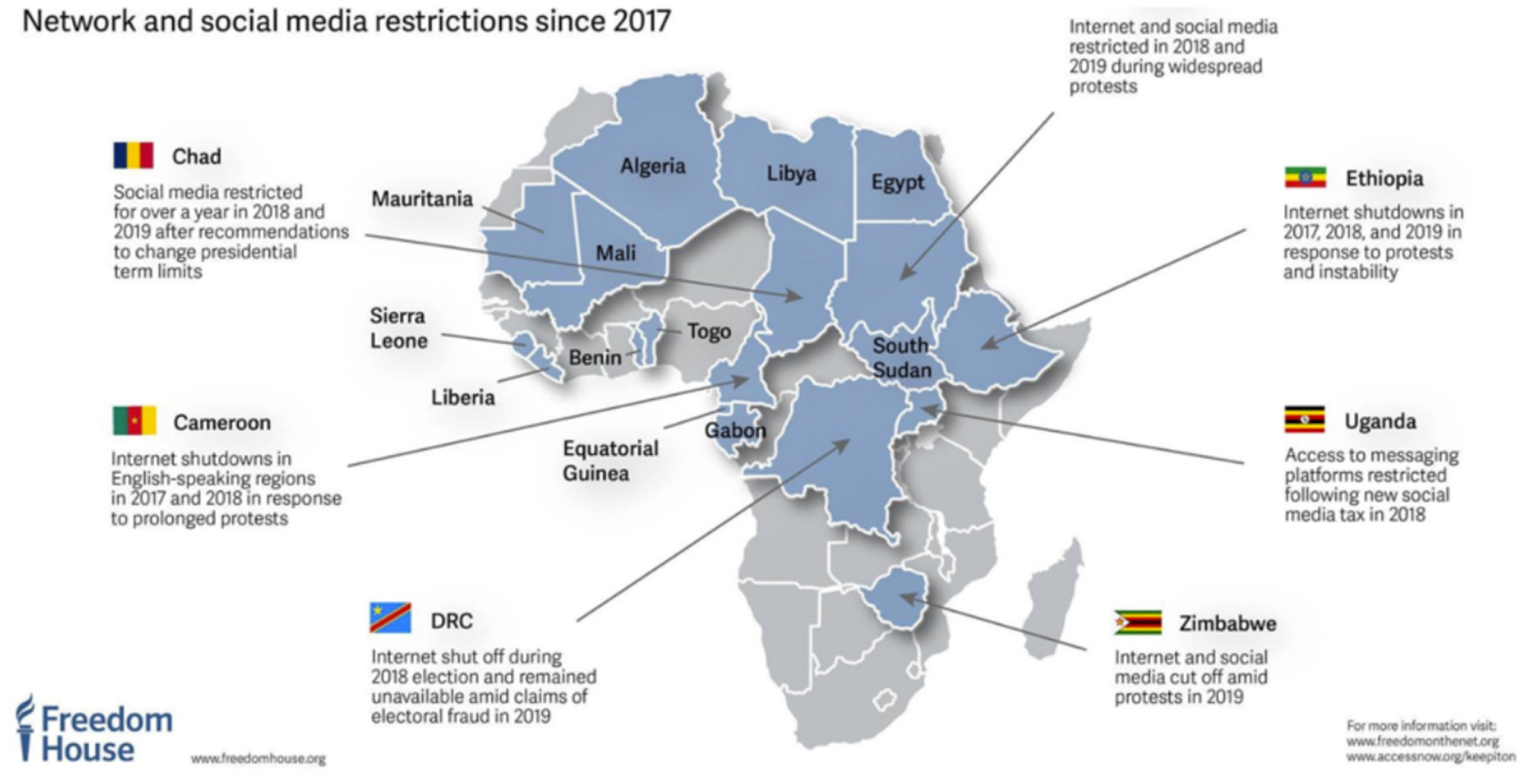
Internet restrictions within Africa between 2017 and 2019 from Freedom House data Available online at: https://twitter.com/freedomhouse/status/1138135355931201536/photo/1 (accessed April 4, 2021).

In addition to sanctions-related geoblocking, there is also an increasing trend for national governments to use access restrictions as a means of political control. As demonstrated in Figure 3 below, a number of African countries have recently experienced limitations on freedom of press and access to information^20^. Similar limits have been reported from other countries across the globe^21^. While not directed at academia, it is evident that these shutdowns can have a significant impact on research within these countries.

### Time-Outs and Last-Mile Connection Issues

While the challenges of overt access restriction are becoming increasingly visible, there are a range of other access issues that are widespread, pernicious and regularly overlooked. These relate to poor connectivity that hampers researchers across the globe. These “last mile” challenges refer to the inaccessibility of online data due to a range of issues including low bandwidth, unstable connectivity, power outages and the cost of data (Bezuidenhout et al., 2017a).

Unstable connectivity can mean that when a web browser makes a request for a web page a sufficient period of time will pass where the browser does not get a response and hence creates a time-out error. This in particular can affect large data downloads or downloads of multiple smaller files.

The work-from-home requirements of the COVID-19 pandemic presented additional challenges to researchers working in many low/middle-income countries (LMICs) due to data transmission costs. For many researchers, landline or fibreoptic connectivity is not possible in their home context, meaning that they relied on mobile data for connectivity. A recent study on mobile data costs demonstrated that the three countries with the most expensive mobile data per 1GB are all in Africa. These were Malawi ($27.41), Benin ($27.22), and Chad ($23.33)^22^. These data costs, when put into perspective with the average salaries of researchers and postgraduate students in these countries, makes accessing datasets or engaging with online collaborations prohibitively expensive.

## Quantifying the Absence of Accessibility from a User Perspective

While the introduction presents a range of concerns relating to the accessibility of data, it is difficult to advocate for action on these issues without quantifiable data. To date, much of the evidence presented in support of these concerns relies on small qualitative studies or anecdotal evidence. In order to address the paucity of data, the authors set out to quantify the level of difficulty associated with getting access this paper simulates access from a range of countries (high, middle and low income; some under sanction and others not) by using web proxies of a set of web sites that are key for Open Science.

Rather than focusing on the content of the pages downloaded, this study set out to test whether sites were downloaded at all and what error codes were returned if there was a failure to download. The results thus do not distinguish between access time-out or blocking. The results were recorded and compared with the results from the other countries in the study.

### Methodology

The analysis is based on two steps. In the first instance a set of web sites were selected that are used in Open Science. Once that list was collated, proxies were set up for a set of countries to examine access to those sites from the set of countries^23^. All of the software developed for this project and accompanying data can be found on the repository Zenodo (Shanahan and Bezuidenhout, 2022).

#### Selection of Suitable Web Sites

Two sets of web sites were collated. In the first instance a curated set of 254 web sites from 101 tools + JISC list of open science tools (Bezuidenhout and Havemann, 2021). This lists key sites such as github.com, bioarxiv.com and osf.io. A second set of web sites was collated from Re3Data which is a registry of research data repositories. A script was run to download all the web sites listed in re3data in June 2020. The URLs and Re3Data IDs for 2527 sites were downloaded using this approach. Hence 2,781 URLs were collated for this study.

#### Proxies

Fourteen countries were selected to download the above total list of URLs. These countries are Cuba (cu), the United Kingdom (gb), Ireland (ie), Iraq (iq), Iran (ir), Japan (jp), North Korea (kp), Myanmar (mm), Sudan (sd), Syria (sy), the United States of America (us), Venezuela (ve), Yemen (ye), and South Africa (za). Their corresponding ISO 3166 standard two letter code^24^ are listed in parenthesis and in this paper these codes will be used.

**Figure 4:**
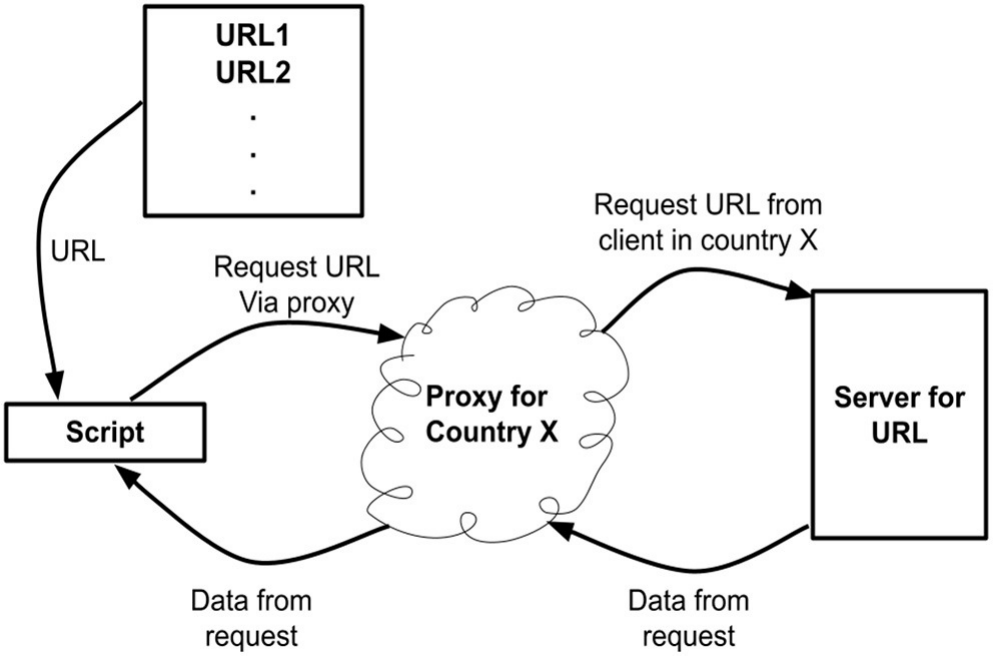
Schematic of software used to download data.

The proxy service provided by the company Bright Data^25^ (previously referred to as Luminati) was used to provide clients in each of the above countries. A schematic of the software can be found in Figure 4. Using the API of Bright Data for each country a request was made to download the URLs in the above list. The User-Agent string for the HTTP request was set to correspond to the most up to date chrome browser^26^. Download data was captured and stored in individual JSON files. This data includes the HTTP response status codes, the HTML downloaded if access was successful and error codes if access was unsuccessful. The data was gathered in August 2020.

## Results

Two types of data collected during the study are presented below. The first attempts to identify the effect of data not being returned because of connectivity issues. In particular we contrast sites where access is unsuccessful in specific countries with the same sites being successful in downloading in other countries. This can be tracked with a timeout error code in the former and a return code of 200 in the latter (which indicates a successful download)^27^. The second looks for potential cases where sites are blocking access for users in specific countries and allowing access for others. In this case one tracks cases which return a code of 403 (described previously) and a 200 code.

### Access to Sites Is Variable Across Countries

**Figure 5:**
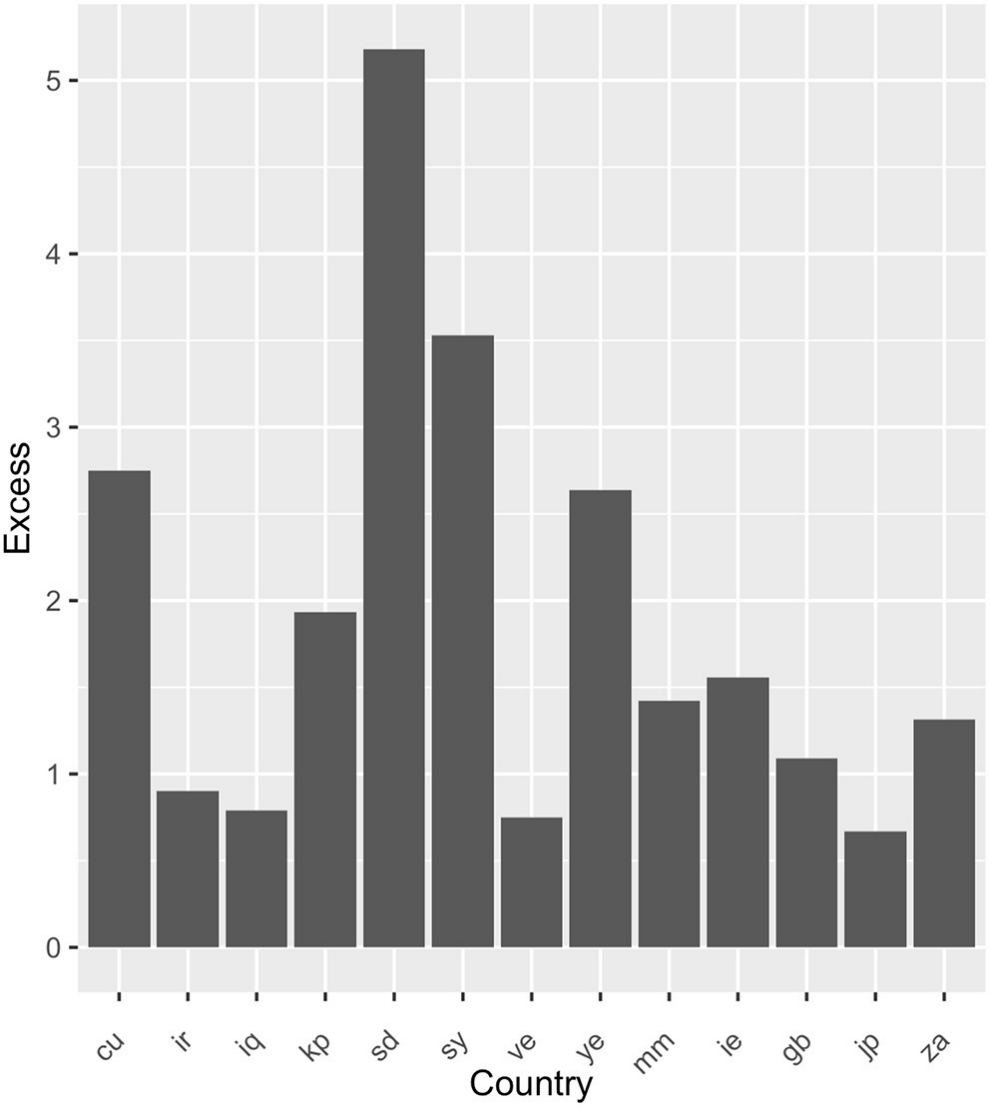
Ratio of number of URLs that did not return HTML in a country to the number of URLs that did not return HTML for us.

For each country the number of sites that did not return any HTML was noted. It is noted that 284 sites consistently could not be downloaded from any country. For each pair of countries (c1,c2c2) the number of sites that failed to return HTML when downloading from c1 but did return HTML when downloaded from c2 was computed. This is referred to as N(c1,c2c2). Assuming that the us will have near-universal access Figure 5 plots the ratio N(c1,c2c2)/N(c2,c1c1) where c1 are the other countries in the list and c2 = us. If two countries are able to access precisely the same sites then the ratio should be one. If c1 can access more sites than c2 then the ratio is <1 and correspondingly if c2 can access more sites than c1 then the ratio should be >1. The results are summarized in Figure 5.

From the above results we find that countries have a variable level of access to the URLs. In particular, Cuba, Sudan, Syria, and Yemen (cu, sd, sy, and ye) are much more likely to be unable to download the URLs. Countries such as Ireland, Japan. and the United Kingdom (ie, jp, and gb) give a range of excess values indicating that the spread of values for Iran, Iraq, Venezuela, and Myanmar (ir, iq, ve, and mm) are not significant. This corresponds to cases where connectivity is poor.

### Specific Blocking of Countries Appears to Exist

**Figure 6:**
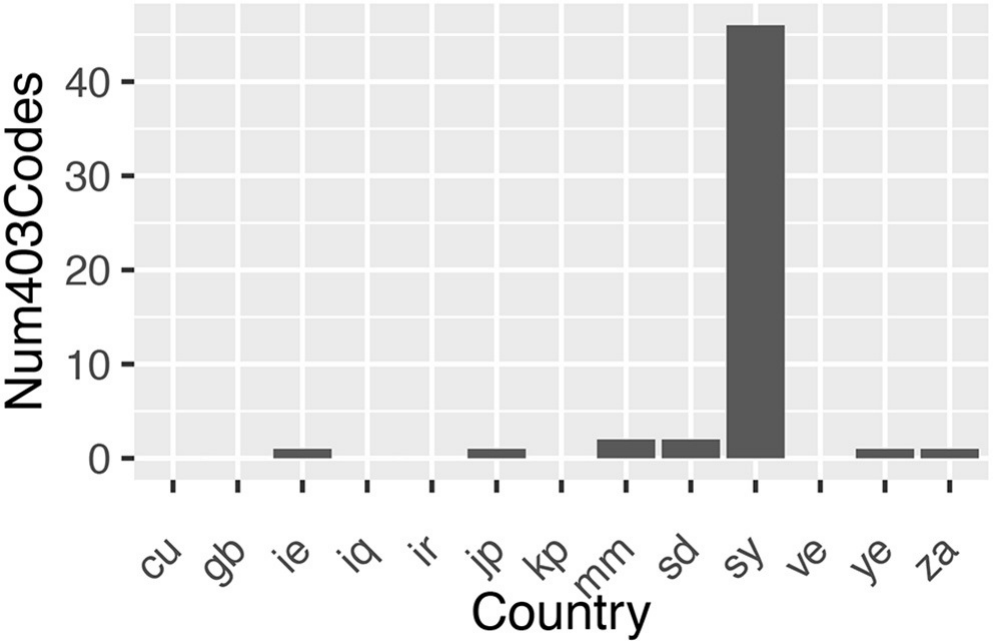
Number of URLs that returned a 403 HTTP response status code when the same URL returned a 200 HTTP response status code from US.

If a web server understands a request to access a URL from a client but refuses to authorize it then it returns a HTTP response status code of 403, as opposed to a response code of 200 if the request is successful. Using US again as a control, for each country the set of URLs which return a 403 response code for that country and returns a 200 response code for US were collated. The number found for each country are plotted in Figure 6. The URLs these correspond to are listed in the Appendix. As evidenced in Figure 6, the significant increase in 403 status codes for Syria (sy) suggests possible geoblocking.

## Discussion

The approach of using proxies to test the access to data is a useful tool for exploring accessibility from a FAIR perspective. Specific geoblocking of sites is harder to detect as some sites may be directly blocked (posting a 403 code) but others, such as Github, may list a web page but may return different content indicating that access to the site is blocked. These preliminary data clearly demonstrate that more research is urgently needed in order to problematize this issue and provide data to inform future Open Science policies. Nonetheless, even this preliminary data raises important issues relating to the accessibility described and defined through the FAIR data principles. These include the observations that:

1. Accessible in-country doesn't mean accessible in all countries
2. User-experiences of FAIR data may vary considerably—as may scores when testing from places that return 403s
3. Discussions on FAIR accessibility cannot be de-coupled from broader discussions on access to Open Data.

### Accessible In-country Doesn't Mean Accessible in All Countries

Figure 1 above illustrates the geographic distribution of trusted digital repositories. It clearly demonstrates that there is a significant bias toward repositories located in HICs. This bias is unsurprising, as HICs continue to dominate global research and development (R&D) expenditure (69.3% in 2013) as well as host the majority of researchers^28^. Because the majority of the repositories, as well as the bulk of their users, are located in HICs, it is possible that this has implications for the “FAIRness” of their design.

The criteria associated with the FAIR accessibility principle means that it is possible for data to be considered accessible without all researchers being able to query data/metadata from their geographic locations. This means that the identifier used to query the database does not return the appropriate data/metadata. It is important to note that this lack of return is likely not related to the standardized communications protocols in place, but rather due to additional barriers in place at various points in the data journey. This draws attention to the possibility that discussions of data accessibility need to be expanded beyond metadata and query protocols to consider a broader range of barriers embedded within the digital landscape.

When considering an expanded discussion around data accessibility it is important to note that there are likely no “quick fixes.” The use of VPNs has been suggested as a tool for bypassing geopolitical barriers to data, as a means of virtually locating the user request in a different country. However, advocating for the use of VPNs as a means of integrating into the current data landscape must raise concerns. Some countries with repressive governments have outlawed VPNs as a means of maintaining control over information flows^29^. Furthermore, national governments have also been reported to engage in VPN blocking as a means of censorship and control^30^. Requiring researchers to use VPNs as a means of engaging with the current Open Science infrastructure can thus place them in positions of personal risk and can thus not be viewed as a viable alternative to the current problem. VPNs will also not fix overall connectivity issues.

### User-Experiences of FAIR Data May Vary Considerably

Even when there are no barriers to accessing the data stored in trusted digital repositories, the evidence presented in this paper suggest that user experiences of interacting with FAIR data may vary considerably around the world. Understanding FAIR from a user perspective is important not only as a means of improving downstream service provision, but also as a means of community engagement. The success of the FAIR principles is contingent on the engagement of researcher communities, and their subsequent adoption of FAIR research practices. If some user communities continue to struggle to access and re-use FAIR data it is possible that this may affect the levels of community engagement and support. Such concerns follow on from similar observations from studies on support for Open Data practices (Bezuidenhout et al., 2017b)^31^. This lack of engagement could lead to a lag in the adoption of FAIR data practices and exacerbate the existing under-representation of certain user communities within the FAIR landscape.

### Discussions on FAIR Accessibility Cannot Be De-coupled From Broader Discussions on Access to Open Data

A central element of current data discussions is the statement that while not all data can be open (e.g., some research data, such as medical data, needs to remain private, and access-controlled), all data must be FAIR. This coupling of Open and FAIR has been used by governments, funders, and institutions to strengthen their commitment to Open Science. As highlighted by Higman, Bangert, and Jones FAIR principles “are being applied in various contexts; the European Commission has put the FAIR principles at the heart of their research data pilot alongside open data. Beyond Europe, the American Geophysical Union (AGU) has a project on Enabling FAIR Data and the Australian Research Data Commons (ARDC) supports a FAIR programme” (Higman et al., 2019, p. 1). Funded researchers are increasingly expected to ensure that the data produced in their research are FAIR, regardless of whether it will be Open.

Within Open Data/Open Science discussions there is a growing recognition that the so-called “digital divide” continues to slow down the evolution of the global research ecosystem. Indeed, infrastructural challenges are regularly mentioned in relation to Open Science in LMICs (CODATA Coordinated Expert Group, 2020) and highlight the need for large-scale infrastructural investment. In contrast, however, similar discussions about local infrastructure are not a priority in FAIR discussions. Not addressing the impact of infrastructures on FAIR-ifying data has a number of consequences. It either suggests that making data accessible is not influenced by the infrastructure available to researchers, or provides the impression that nothing can be done *at the moment* by individual researchers until the research infrastructures evolve.

## Final Comments

It is recognized that data FAIRness is a “moving target” and as infrastructure, practices and processes continue to develop so too will the requirements of what is regarded as being sufficiently FAIR. This awareness reflects the nature of the FAIR principles, namely that they are aspirational (i.e., they are not a set of well-defined technical standards) and do not strictly define how to achieve a state of “FAIRness.” As described by Wilkinson and colleagues, the FAIR data principles “describe a continuum of features, attributes, and behaviors that will move a digital resource closer to that goal” (Wilkinson et al., 2018, p. 1). This ambiguity, they suggest, has led to a wide range of interpretations of what constitutes a FAIR resource^32^.

The ambiguity of what FAIRness constitutes can be thought of on many different levels, but underpins the non-absoluteness of the concept. This paper advocates for the further discussion on how the FAIR principles are translated into action. In contrast to current discussions that focus on the interpretation of the FAIR principles from a disciplinary perspective, this paper emphasizes the urgent need for discussions on the variability introduced by *geographic* and *geo-political factors*. In particular, the paper advocates for a critical reflection on the “frames of reference” used as a basis for discussions on what constitutes “as FAIR as possible for the present.” The use of the accessibility principle to illustrate these points is important, as findability and accessibility are widely considered to be the “easier” of the FAIR principles to achieve.

A brief survey of the current geo-political climate around the world suggests that issues relating to accessibility that are raised in this paper are poised to get worse if nothing is done. The current war in Ukraine and the proposed sanctions on Russia by NATO nations suggest that issues of geoblocking might be exacerbated going forward^33^. Issues of access and time-outs are becoming more frequent due to a growing trend of using internet access to control civil unrest. Moreover, the investment in information and communication technologies in LMICs, while growing, will continue to present challenges for decades to come. Researchers in these regions are unlikely to experience a “level playing field” of connectivity with their HIC colleagues for decades.

Bringing these often-overlooked issues together highlights how current FAIR discussions on data accessibility often fail to recognize pressing challenges experienced by many researchers around the world. To date, there has been little recognition of these issues, let alone discussion of responsibilities for addressing these issues. It is anticipated that any attempts to rectify the current situation will require a joint effort from the international research community, national governments and international data organizations.

As the research landscape continues to evolve through the creation of national and regional Open Science Clouds, these issues are timely. The evolution of FAIR discussions to include principles such as TRUST should serve to further foreground these issues. Indeed, the TRUST principles commit repositories to Monitoring and identifying evolving community expectations and responding as required to meet these changing needs (Lin et al., 2020, p. 3). Recognizing the issues of accessibility means that understandings of what constitutes a “community” need to be critically unpacked. Indeed, the considerable heterogeneity of research communities around the world, and the challenges that they face, needs to be better addressed within FAIR/TRUST discussions, as well as integrated into the technical design of the evolving Open Science landscape.

## Data Availability Statement

The datasets presented in this study can be found in online repositories. The names of the repository/repositories and accession number(s) can be found at: 10.5281/zenodo.6411335.

## Author Contributions

Both authors listed have made a substantial, direct, and intellectual contribution to the work and approved it for publication.

## Conflict of Interest

LB was employed by DANS (Data Archiving and Networked Services). The remaining author declares that the research was conducted in the absence of any commercial or financial relationships that could be construed as a potential conflict of interest.

## Publisher's Note

All claims expressed in this article are solely those of the authors and do not necessarily represent those of their affiliated organizations, or those of the publisher, the editors and the reviewers. Any product that may be evaluated in this article, or claim that may be made by its manufacturer, is not guaranteed or endorsed by the publisher.

## Footnotes

^1^https://www.force11.org/group/fairgroup/fairprinciples (accessed April 4, 2021).

^2^https://fairsfair.eu/ (accessed April 4, 2021).

^3^https://www.coretrustseal.org/ (accessed April 4, 2021).

^4^https://www.coretrustseal.org/why-certification/coretrustsealfair-statement-of-cooperation-support/ (accessed April 4, 2021).

^5^www.fairsfair.eu/f-uji-automated-fair-data-assessment-tool (accessed April 4, 2021).

^6^www.fair-enough.semanticscience.org (accessed April 4, 2021).

^7^www.fair-checker.france-bioinformatique.fr/base_metrics (accessed April 4, 2021).

^8^www.adrc.edu.au/resources/aboutdata/fair-data/fair-self-assessment-tool/ (accessed April 4, 2021).

^9^www.fairsfair.eu/fair-aware (accessed April 4, 2021).

^10^https://ec.europa.eu/info/research-and-innovation/strategy/strategy-2020-2024/our-digital-future/open-science/open-science-monitor_en (accessed April 4, 2021).

^11^www.re3data.org (accessed April 4, 2021).

^12^https://www.re3data.org/browse/by-country/ (accessed April 4, 2021).

^13^https://digital-strategy.ec.europa.eu/en/news/geo-blocking-regulation-questions-and-answers (accessed April 4, 2021).

^14^https://github.blog/2021-01-05-advancing-developer-freedom-github-is-fully-available-in-iran/ (accessed April 4, 2021).

^15^https://www.w3.org/TR/geolocation/ (accessed April 4, 2021).

^16^https://httpd.apache.org (accessed April 4, 2021).

^17^https://developer.mozilla.org/en-US/docs/Web/HTTP/Status#client_error_responses (accessed April 4, 2021).

^18^https://www.bbc.com/news/business-56685136 (accessed April 4, 2021).

^19^https://www.stripes.com/news/us/navy-pulls-plug-on-climate-task-force-after-pentagon-deems-climate-change-a-national-security-issue-1.596184#.XWYgtXfMVhg.twitter (accessed April 4, 2021).

^20^https://blogs.lse.ac.uk/africaatlse/2020/05/28/popular-support-for-media-freedom-press-africa-complicated-picture/ and https://www.bbc.com/news/world-africa-59958417 (accessed April 4, 2021).

^21^https://twitter.com/Meenwhile/status/1265711539140440064 and https://netblocks.org/ and https://www.bbc.com/news/world-asia-55923486 (accessed April 4, 2021).

^22^https://www.visualcapitalist.com/cost-of-mobile-data-worldwide/ (accessed April 4, 2021).

^23^The methodology was based on a paper by McDonald et al. that focused on geoblocking (McDonald et al., 2018).

^24^https://www.iso.org/iso-3166-country-codes.html (accessed April 4, 2021).

^25^https://brightdata.com (accessed April 4, 2021).

^26^Chrome/84.0.4147.89 (accessed April 4, 2021).

^27^https://developer.mozilla.org/en-US/docs/Web/HTTP/Status#successful_responses (accessed April 4, 2021).

^28^https://en.unesco.org/node/252279 (accessed April 4, 2021).

^29^https://protonvpn.com/blog/are-vpns-illegal/ (accessed April 4, 2021).

^30^https://en.wikipedia.org/wiki/VPN_blocking (accessed April 4, 2021).

^31^https://ec.europa.eu/info/research-and-innovation/strategy/strategy-2020-2024/our-digital-future/open-science/open-science-monitor/facts-and-figures-open-research-data_en#funderspolicies (accessed April 4, 2021).

^32^An analogy can be drawn with physical infrastructure. A rope bridge and suspension bridge both enable traversal of a river. One may regard the latter as a better implementation overall for doing (more stable, able to carry greater loads) but the former may well be an excellent starting point as it is low cost and can be set up quickly.

^33^https://www.bbc.com/news/world-europe-60125659 (accessed April 4, 2021).

## Appendix

**Table A1:** URLs that returned a 403 response code for a given country (returning 200 for us).

**Country code**	**URLs receiving a 403 code**
ie	www.hon.ch/HONmedia/
jp	https://www.census.gov/geo/maps-data/data/tiger-line.html
mm	• https://www.census.gov/ • https://dnr.mo.gov/geology/geosrv/
sd	• https://data.mendeley.com/ • https://www.mendeley.com/
sy	• figshare.com
	• forschung.deutsche-rentenversicherung.de/FdzPortalWeb
	• ds.data.jma.go.jp/gmd/wdcgg
	• www.nothobranchius.info
	• geodata.grid.unep.ch
	• geocommons.com
	• geogratis.cgdi.gc.ca
	• cbeo.communitymodeling.org
	• henke.lbl.gov/optical_constants
	• hubblesite.org/gallery
	• www.mitime.org/mirage
	• iobis.org
	• jaspar.genereg.net
	• kpbc.umk.pl
	• wdcpc.org
	• nfdp.ccfm.org
	• neuromorpho.org
	• nfdp.ccfm.org
	• patterns.projects.cis.ksu.edu
	• qed.econ.queensu.ca/jae
	• ccdb.wishartlab.com
	• repository.up.ac.za
	• www.kadonis.org
	• wwwdev.ebi.ac.uk/pride
	• portal.clarin.nl
	• vsso.cssdc.ac.cn
	• clarin.informatik.uni-leipzig.de/repo
	• wals.info
	• wdc.dlr.de
	• sedac.ciesin.columbia.edu
	• simbad.u-strasbg.fr/simbad
	• slgo.ca/en
	• westnile.ca.gov
	• uk-air.defra.gov.uk/data
	• www.bgs.ac.uk/services/ngdc
	• www.bmrb.wisc.edu
	• www.brc.ac.uk
	• www.caida.org/data
	• www.calsurv.org
	• www.cdc.gov/DataStatistics
	• www.forestdata.cnttp://www.chemspider.com
	• www.chemsynthesis.com
	• climate.weather.gc.ca
	• www.crystallography.net/cod
	• www.afdc.energy.gov
ye	www.runmycode.org
za	https://www.census.gov/
